# Audit and feedback and clinical practice guideline adherence: Making feedback actionable

**DOI:** 10.1186/1748-5908-1-9

**Published:** 2006-04-28

**Authors:** Sylvia J Hysong, Richard G Best, Jacqueline A Pugh

**Affiliations:** 1 Houston Center for Quality of Care and Utilization Studies, Michael E. DeBakey VA Medical Center, Houston, Texas, USA; 2 Department of Medicine – Health Services Research Section, Baylor College of Medicine, Houston, Texas, USA; 3 Healthcare Solutions Division, Lockheed Martin Information Technology, San Antonio, Texas, USA; 4 Veterans Evidence-Based Research Dissemination and Implementation Center, South Texas Veterans Health Care System, San Antonio, Texas, USA; 5 Department of Medicine, University of Texas Health Science Center at San Antonio, San Antonio, Texas, USA

## Abstract

**Background:**

As a strategy for improving clinical practice guideline (CPG) adherence, audit and feedback (A&F) has been found to be variably effective, yet A&F research has not investigated the impact of feedback characteristics on its effectiveness. This paper explores how high performing facilities (HPF) and low performing facilities (LPF) differ in the way they use clinical audit data for feedback purposes.

**Method:**

Descriptive, qualitative, cross-sectional study of a purposeful sample of six Veterans Affairs Medical Centers (VAMCs) with high and low adherence to six CPGs, as measured by external chart review audits.

One-hundred and two employees involved with outpatient CPG implementation across the six facilities participated in one-hour semi-structured interviews where they discussed strategies, facilitators and barriers to implementing CPGs. Interviews were analyzed using techniques from the grounded theory method.

**Results:**

High performers provided timely, individualized, non-punitive feedback to providers, whereas low performers were more variable in their timeliness and non-punitiveness and relied on more standardized, facility-level reports. The concept of actionable feedback emerged as the core category from the data, around which timeliness, individualization, non-punitiveness, and customizability can be hierarchically ordered.

**Conclusion:**

Facilities with a successful record of guideline adherence tend to deliver more timely, individualized and non-punitive feedback to providers about their adherence than facilities with a poor record of guideline adherence. Consistent with findings from organizational research, feedback intervention characteristics may influence the feedback's effectiveness at changing desired behaviors.

## Background

Audit and feedback (A&F) has been used for decades as a strategy for changing the clinical practice behaviors of health care personnel. In clinical practice guideline (CPG) implementation, A&F has been used to attempt to increase guideline adherence across a wide variety of settings and conditions, such as inpatient management of chronic obstructive pulmonary disease (COPD)[1], test ordering in primary care[2,3], and angiotensin-converting enzyme (ACE) inhibitor and beta-blocker usage in cardiac patients[4]. Recent reviews, however, indicate that the effectiveness of A&F as a strategy for behavior change is quite variable. Grimshaw and colleagues[5] reported a median effect size of A&F of +7% compared to no intervention using dichotomous process measures, with effect sizes ranging from 1.3% to 16%; however, that same review reported non-significant effects of A&F when continuous process measures were used. Along similar lines, Jamtvedt and colleagues [6] reported a median adjusted relative risk of non-compliance of .84 (interquartile range (IQR): .76–1.0), suggesting a performance increase of 16% (IQR: no increase to 24% increase). Such studies attribute much of the variability in effect of the interventions to (often unrecognized) differences in the characteristics of the feedback used in the intervention and/or to the conditions under which A&F is more likely to be effective [6-9].

Earlier A&F research has suggested that the timing of feedback delivery can influence the resulting behavior change[10], as can the credibility of the feedback source [11-13]. Research from the organizational literature suggests a host of other potential explanatory phenomena as potentially affecting the effectiveness of feedback, such as its format (e.g., verbal vs. written), its valence (i.e., whether it is positive or negative)[14], and its content (e.g., whether it is task-focused or person-focused, individual or group based, normative or ipsative)[15]. Our own research has noted that facilities with higher CPG adherence (i.e., high performing facilities, or HPF) relied more heavily on chart data as a source of feedback and placed greater value on educational feedback approaches than facilities with lower guideline adherence (low performing facilities, or LPF)[16]. Taken together, these research findings indicate a need to further explore the characteristics of A&F and their impact on the desired behavioral change. Building on our previous work on barriers and facilitators of clinical practice guideline implementation, the purpose of the analyses reported here is to address this need in the A&F literature by exploring how HPF and LPF differ in the way they use clinical audit data for feedback purposes.

## Methods

### Measurement of clinical practice guideline adherence

Guideline adherence was measured via External Peer Review Program (EPRP) rankings. EPRP is a random chart abstraction process conducted by an external contractor to audit performance at all VA facilities on numerous quality of care indicators, including those related to compliance with clinical practice guidelines. We obtained data for fiscal year 2001 reflecting facility-specific adherence to guideline recommendations for six chronic conditions usually treated in outpatient settings: diabetes, depression, tobacco use cessation, ischemic heart disease, cardiopulmonary disease, and hypertension. Each condition is monitored via multiple performance indicators; in total, 20 performance indicators were used to describe compliance across the six conditions. Facilities were rank ordered from 1–15 (15 being the highest performer) on each performance indicator. HPF tended to rank consistently high across most disease conditions, and LPF tended to consistently rank low across most disease conditions; consequently, all 20 performance indicator ranks were summed to create an indicator rank sum (IRSUM) score [higher IRSUM scores indicate higher performance]. Facilities then were rank-ordered according to their IRSUM score to identify the three highest and the three lowest performing facilities, which were used for sample selection.

### Site selection

The data herein were part of a larger data collection effort at 15 VA facilities designed to examine barriers and facilitators to CPG implementation[17]. These facilities were selected from four geographically diverse regional networks using stratified purposive sampling. To be invited to participate, facilities had to be sufficiently large to accommodate at least two primary care teams, each containing at least three MD providers. In order to address the present paper's specific research question, only the highest and lowest performing facilities (based on their IRSUM score described above) were included in the sample. Thus, the final sample for this paper consisted of employees at three HPF and three LPF.

### Participants

One-hundred and two employees across six facilities were interviewed. Within each facility, personnel at three different organizational levels participated: Facility leadership (e.g., facility director, chief of staff), middle management and support management (e.g., quality assurance manager, primary care chief, information technology manager), and outpatient clinic personnel (e.g., physicians, nurses, and physicians' assistants). All three levels were adequately represented in the sample (see Table 1). No significant differences in the distribution of participants were found by facility or organizational level (χ^2^_10 _= 17.4, n.s.). Local contacts at each facility assisted in identifying clinical and managerial personnel with the requisite knowledge, experience, and involvement in guideline implementation to serve as potential participants. The study was locally approved by each facility's institutional review board (IRB), and participation at each facility was voluntary. An average of nine interviews occurred at each facility, for a total of 54 interviews at the six facilities (Table 1).

**Table 1:** Number of participants by facility and hierarchical level

***Facility***		***Hierarchical Level***			***Total # of Participants***	***Total # of Interviews***
				
		Primary Care Personnel	Middle/Support Management	Facility Leadership		
High Performers	1	14	2	3	19	8
	2	6	10	7	23	14
	3	7	4	3	14	9

Low Performers	4	4	8	4	16	8
	5	3	4	4	11	7
	6	7	10	2	19	8

	Total	41	38	23	102	54

Note: Facilities are listed in decreasing order of performance. No significant differences in the distributions of participants were found by facility or hierarchical level (χ^2^_10 _= 17.4, n.s.).

### Procedure

Three pairs of interviewers were deployed into the participating sites during the spring of 2001. The interviewers were research investigators of various backgrounds (e.g., medicine, nursing, organizational psychology, clinical psychology, and sociology), with in-depth knowledge of the project, and most were involved with the project since its inception. None of the interviewers was affiliated with any of the participating facilities.

Each pair travelled to a given site for two days, where together the interviewers conducted one-hour, semi-structured interviews either individually or in small groups, depending on the participants' schedule and availability (see appendix for interview guide and protocol). Interviewers took turns leading the interview, while the secondary interviewer concentrated on active listening, note-taking, and asking clarifying questions. Interviewers discussed their own observations after each interview, and compiled field notes for each facility based on these observations and discussions. To minimize interviewer bias, interviewer pairs were (a) blinded to the facility's performance category, and (b) split and paired with different partners for their following site visit. All interviewers were trained *a priori *on interviewing and field note protocol.

Participants were asked how CPGs were currently implemented at their facility, including strategies, barriers and facilitators. Although interviewers used prepared questions to guide the interview process, participants were invited to (and often did) offer additional relevant information not explicitly solicited by the interview questions. The interviews were audio recorded with the participants' consent for transcription and analysis.

### Data analysis

Interview transcripts were analyzed using a grounded theory approach[18,19]. Grounded theory consists of three analytic coding phases: open, axial, and selective coding – each is discussed below. Transcripts were analyzed using Atlas.ti 4.2, a commonly used qualitative data analysis software program[20].

#### Open coding

Automated searches were conducted on the interview transcripts for instances of the following terms: "feedback," "fed back," "feeding back," "report" and its variations (e.g., reporting, reports, reported), "perform" and its variations (e.g., performing, performed, performance), "audit" and its variations (e.g., auditing, audited, audits), and "EPRP". All word variations were captured via a truncated word search. The results were then manually reviewed for relevance, and only passages that specifically discussed feedback on individuals' adherence to clinical practice guidelines were included. Examples of excluded feedback references included feedback about the computer interface to information technology personnel, or anecdotal comments received from patients about provider adherence. This review resulted in 122 coded passages across the 54 interviews in the six facilities, for an average of 20 coded passages per facility.

#### Axial coding

In this phase of analysis, the passages identified during open coding are compared and thematically organized and related. This process resulted in identification of four characteristics of feedback from the data: timeliness, individualization, customizability and punitiveness. Each is discussed in more detail in the results section. Passages identified during open coding were categorized among these four properties and were organized by facility according to each of these properties. To ensure coding quality and rigor, code definitions were explicitly documented as soon as they emerged, and were continuously referred to throughout the coding process. Code assignment justifications were written for each passage as it was categorized, and coded passages were re-examined to insure that code assignments were consistent with code definitions. Patterns in the high performing facilities were compared among each other, searching for potential commonalities, as were patterns in the low-performing facilities. Once patterns were identified we relied on the corpus of field notes and informal observations from interviewers to provide interpretive context.

#### Selective coding

This phase of analysis involves integrating and refining the ordered categories from the axial coding phase into a coherent model or theory, usually based on a core or central category from the data. Based on the pattern of passages examined during axial coding, the "customizability" category emerged as the critical phenomenon around which a model grounded in the data was constructed, centering on the concept of actionable feedback. This is discussed in more detail in the results section.

## Results

### Feedback characteristic patterns in high and low performing facilities

Four characteristics emerged from the data that described the nature of feedback received by clinicians at VA outpatient facilities. Table 2 summarizes the patterns of feedback use across the six facilities. Each characteristic is discussed in more detail below.

**Table 2:** Patterns of feedback properties by facility

	**High Performers**			**Low Performers**		
	
**PROPERTY**	1	2	3	4	5	6
Timely	E	E	E	E	C	C
Individualized	E	E	C	N	N	N
Non-Punitive	E	E	I	I	N	I
Customizable	I	I	I	N	N	I

Note: E = Evidence was observed that the property in question was present at that facility; C = conflicting evidence; I = insufficient evidence; and N = negative evidence, i.e., evidence that the opposite property was present (e.g., an N for facility 4 on individualized means that there is evidence that the feedback is not individualized).

#### Timeliness

This refers to the frequency with which providers receive feedback. Monthly or more frequent feedback reports were considered timely; quarterly or less frequent reports were considered untimely. We chose monthly feedback as the timeliness threshold because, given usual time intervals between appointments within VA, quarterly or less frequent feedback may not give the provider sufficient time to change his/her behavior in time for a patient's next appointment.

All facilities reported delivering feedback in a timely manner. However, as seen in Table 2, the evidence for timeliness of feedback is more mixed in the low-performing facilities than in the high-performing facilities. Conflicting reports of timely and untimely feedback delivery were observed in the low-performing facilities, whereas timely feedback delivery was clearly the dominant practice in high-performing facilities (all names and initials in quotations are fictitious, to protect participant confidentiality):

*And then we also do what's basically called, excuse me, provider score cards for the VISN's, and it will show exactly in which areas they were found lacking throughout the entire process for all the CPG's, for all the PI's [performance indicators]. .... Q: And that's how often? A: About once a month*.

*-- R.R., a support management employee in a HPF*.

#### Individualization

This refers to the degree to which providers receive feedback about their own individual performance, as opposed to aggregated data at the team, clinic or facility level. As can be seen from the table, none of the low-performing facilities provided individualized feedback to their providers. In most cases, individual providers received facility level data from the EPRP report.

*To be honest, most of the monitoring has really been done through the EPRP data collection. If one looks at some of the other guidelines, such as our COPD guideline, there we really don't have a formal system set up for monitoring that. So if one really looks at performance and outcomes, EPRP remains probably our primary source of those types of data*.

*-- B.F., An executive level employee in a LPF*.

In contrast, all three high-performing facilities reported providing individual level data to their providers:

*Feeding it back, the individual reports go back to the practitioners and providers so they would see for a specific patient that was reviewed for where actual outcomes were. And many of them take that information to heart and would actually look, go back to the medical records and say, "Oh yeah! You're right! I missed this," or "Oh no! You guys didn't pick up this." And they'd go back and show us where they documented it and that would allow us to have the dialogue*.

*-- M.M., a support management employee in a HPF*.

#### Punitiveness

This concerns the tone with which the feedback is delivered. Two out of the three HPF explicitly reported that they approached underperforming providers in a non-punitive way to help them achieve better adherence rates.

It's a little more than that. She [the chief of staff] sends out positive letters. She sends out suggestions for improvement letters. But at the same time the people on the provider fields know that Tom and I both do this review, and I've offered many, many times to say if you've got a case that you don't understand why this didn't meet criteria, call me and we'll look at it together. And I think that's been a real positive for this place because if I can go over that particular case that applies to you, it's much more beneficial

*-- G.D., a support management employee in a HPF*.

*Oh yeah, by provider, by clinic, we track them by clinic. We can tell who, and we don't use it punitively. We just say, we had one provider in particular that was not doing very well. And we just showed him data, and "this is your comparative data" and all your other providers in the clinic are getting this done. And why are you not? And he's like, "thank you for telling me," and he jumped up there and is doing as well as everybody else*.

*-- M.B., a support management employee in a HPF*.

In contrast, employees at one LPF made explicit mention of the punitive atmosphere associated with low guideline adherence rates.

Sometimes I almost thought that it was in the overall presentation. If it wasn't so threatening and if it was interactive, and if it was, you can show me and we're going to work with you ... then you can get a better buy-in than you can if just saying, this is it. Do it! Heads will roll! We'll chop off one finger and then we'll go for a hand and a foot, kind of thing!

*-- C.C., a clinician in a LPF*.

*We're down here in the trenches and if something goes wrong, somebody pounds on our head. Otherwise, they leave us alone*.

*-- A.B., a clinician in a HPF*.

For the rest of the facilities, however, there were insufficient reports in either direction to indicate the presence of a punitive or non-punitive approach to delivering feedback.

#### Customizability

This referred to the ability to view performance data in a way that was meaningful to the individual provider. No facilities reported having customizable reports or tools that allowed individual providers to customize their performance information to their needs. Some facilities, however, did report having some capability to customize (even though that capability was not being employed), as expressed by this respondent:

*Yes, we could pull out, let's say, I could pull out all of the patients that have a reminder due with the diabetic foot that's a diabetic. And then I could see that two [providers] have 500 [patients with a reminder due]. You only have 100. Guess who's doing much better. ... My reminder program can do that*.

*-- H.S., a support management employee in a HPF*.

*I've got my computer setup where I can just plug in the numbers, get a new set of numbers, and then update my overall cumulative scores within 10, 15 minutes. And that's what gets fed back very, very quickly*.

*-- S.M., a clinician in a HPF*.

These reports came exclusively from high-performing facilities; however, there were several reports, both from HPF and LPF, about the utility and desirability of having such information.

### A model of actionable feedback

**Figure 1:**
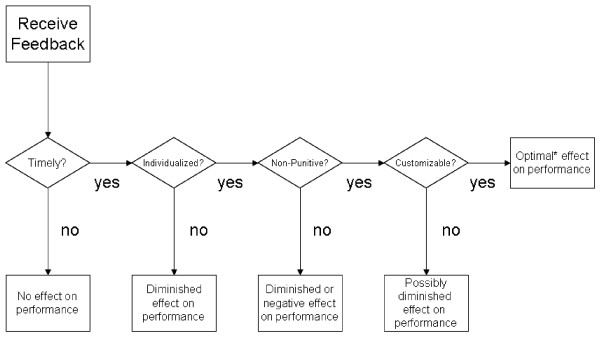
A Model of Actionable Feedback. *The use of the term optimal to describe the effect on performance is relative – by this we mean optimal, given the variables in the emergent model. There are certainly other factors which could affect performance, although they are not exhibited here.

From the pattern of the feedback properties, a hierarchical ordering can be postulated to arrive at a model of actionable feedback (see Figure 1). At a minimum, feedback must be timely in order to be useful or actionable – one can easily imagine situations where the most thoughtful, personalized information would be useless if it were delivered too late. Next, feedback information must be about the right target. In this case, since clinical practice guideline adherence is measured at an individual level (i.e., the data from which adherence measures are constructed concern individual level behaviors such as ordering a test or performing an exam), clinician feedback should be about their individual performance rather than aggregated at a clinic or facility level to maximize its effectiveness[21,22]. Third is non-punitiveness – feedback delivered in a non-punitive way is less likely to be resisted by the recipient regardless of content [15,23,24], thus making it more actionable. Finally, customizability engages the individual with the data, making him/her an active participant in the sense-making process, rather than a passive recipient of information. The proposed hierarchical ordering is reflected in the data. As seen in table 2, four out of six facilities reported using EPRP data to deliver timely feedback to their providers. The HPF provided individualized feedback to their providers, whereas the LPF indicated that they used facility level, rather than provider-specific reports as a feedback source. Only the top two performing facilities specifically indicated that they approached feedback delivery non-punitively, whereas no evidence of this existed either way in the other facilities (save for one LPF which reported explicit instances of punitive feedback delivery). No facilities reported providing their clinicians with the ability to customize their own individual performance data, although all facilities expressed a desire for this capability. Thus, as we move up the facility rankings from the lowest to the highest performer, more of the properties appear to be present. This hierarchical ordering thus leads us to postulate the underlying dimension of "actionable feedback."

## Discussion

We employed a qualitative approach to study differences in how high- and low-performing facilities used clinical audit data as a source of feedback. HPF delivered feedback in a timely, individualized, and non-punitive manner, whereas LPF were more variable in their timeliness, and relied on more standardized facility-level reports as a source of feedback, with one facility reporting a punitive atmosphere. The concept of actionable feedback emerged as the core category in these data, around which timeliness, individualization, non-punitiveness, and customizability can be hierarchically ordered.

The emergent model described above is consistent with existing individual feedback theories and research. Feedback intervention theory (FIT)[15] posits that in order to have a positive impact on performance, feedback should be timely, focused on the details of the task, particularly on information that helps the recipient see how his/her behavior should change to improve performance (correct solution information), and delivered in a goal-setting context. These propositions are consistent with empirical research. Timely feedback has long been positively associated with feedback effectiveness in the organizational literature,[13] as has the need for individualized feedback[21,22]. Feedback delivered in a non-punitive way has been empirically linked to increased likelihood of feedback acceptance[25], a critical moderator of the relationship between feedback and performance[26]. Finally, although the effect of customizable feedback on feedback acceptance and subsequent performance has not been directly examined in the literature, this relationship can be inferred from related research and theory. Research indicates that clinicians want to access and interact with computerized clinical data more naturally and intuitively than is currently offered by EMR systems[27]. FIT proposes that feedback interventions that direct attention toward task details tend to improve performance. The ability of the provider to customize his or her specific performance data into something that is meaningful to him/her is likely to direct attention to the details of the performance measure in question, thereby increasing the likelihood of subsequent performance improvements. This research has implications for both research and practice. First, it suggests that A&F is not an all-or-nothing intervention: how feedback is delivered plays an important role in its effectiveness. Thus, some of the mixed findings in the A&F literature[5,6] could be partially explained by differences in feedback characteristics. Future research should consider such characteristics when designing A&F interventions.

Second, from a practice perspective, this research reminds administrators that A&F, whether for administrative or developmental purposes, is more than simple reporting of performance data. Feedback needs to be meaningful in order for recipients to act on it appropriately. Electronic tools such as VA's Computerized Patient Records System (CPRS) can help provide clinicians timely, individualized and customizable feedback – if used correctly. For example, CPRS is capable of generating individualized, customized reports, however, this capacity is not widely known, and thus remains underused. VA is already taking steps to make this capability better understood, with a re-engineering of CPRS to make template creation and report generation a simpler task for the user, and by offering training on the use of these tools system-wide[28]. However, whether feedback is punitively delivered is strictly a human matter; administrators should take care to adopt an educational, developmental perspective to feedback delivery. All of this, of course, assumes that the data fed back to the clinician are valid and reliable. Issues of sample size (whether sufficient cases of a given indicator exist to calculate a stable estimate for an individual provider), reliability, and appropriateness of behaviours and outcomes as indicators of quality (e.g., Does the clinician really have the power to control a patient's blood pressure level if the patient consistently refuses to follow his/her plan of care?) should be carefully considered when developing and selecting behaviours and outcomes as indicators of clinician performance for feedback purposes.

### Limitations

First, the study's relatively small sample size of six facilities, three in each performance condition, potentially limits the transferability of our results. VA facilities tend to be highly variable across multiple dimensions, and thus this study's findings might not apply to other VA facilities, or to outpatient settings outside the VA. However, two features of this research make us guardedly optimistic about the transferability of the findings. The six sites varied significantly by size, geography, facility type (i.e., tertiary vs. general medicine and surgery), and primary care capabilities; this variation did not significantly differ between HPF and LPF. The presence of a pattern of feedback characteristics, despite the variability in site characteristics, supports the idea that this pattern may be transferable to other facilities. Additionally, the feedback characteristics emergent from the data are consistent with existing research and theory on feedback characteristics, which suggests that our model could be transferable not only to other VA clinics, but potentially to other outpatient settings as well.

Second, the density of reports (20 passages per facility) is somewhat low, which potentially limits the credibility of the findings. However, participants were not explicitly interviewed on the subject of performance feedback, but rather on more general strategies and facilitators of clinical practice guideline implementation. Given the large domain of other available strategies and facilitators that participants mentioned[29], the consistency with which the feedback theme repeats itself across the six facilities strengthens the credibility of these findings, despite the low report density.

Finally, although the emergent feedback characteristics were consistent with previous research, we did not review or validate our findings with the study participants, as data collection and analysis did not occur concurrently. This is an inherent limitation of secondary data analysis and of our reliance on data collected to gain insight into the facilities' CPG implementation strategies and barriers rather than feedback characteristics. Future research should consider both qualitative and quantitative replication of the model.

## Conclusion and future directions

We conclude that facilities with a record of successful guideline adherence tend to deliver more timely, individualized, and non-punitive feedback to providers about their individual guideline adherence than facilities with a poor record of guideline adherence. Consistent with organizational research, feedback characteristics may influence the feedback's effectiveness at changing desired behaviors. Future research should more fully explore the nature and effects of feedback characteristics on their effectiveness in clinical settings, the utility of customizing clinical audit data so that it is meaningful to individual providers, and the effects of meaningful feedback on subsequent performance, especially in comparison to or conjunction with a financial incentive or similar pay-for-performance arrangement. Meanwhile, administrators should take steps to improve the timeliness of individual provider feedback, and deliver feedback from a perspective of improvement and professional development rather than one of accountability and punishment for failure.

## Abbreviations

CPG – Clinical Practice Guidelines

CPRS – Computerized Patient Records System

EPRP – External Peer Review Program

FIT – Feedback Intervention Theory

HPF – High-Performing Facilities

IQR – Inter-quartile Range

LPF – Low-Performing Facilities

OQP – Office of Quality and Performance

VA – Veterans Affairs

VAMC – Veterans Affairs Medical Center

## Competing interests

The research reported here was supported by the Department of Veterans Affairs, Veterans Health Administration, Health Services Research and Development Service (HSR&D) (CPI #99–129). All three authors' salaries are supported, in part, by the Department of Veterans Affairs. The authors declare they have no other competing interests, financial or non-financial.

## Authors' contributions

SH interviewed participants, coded interview transcripts, and was principally responsible for the research idea, design, analyses, and drafts of this manuscript. RB was involved in all aspects of the study, including project management, participant interviews, coding interview transcripts, and editing of manuscript drafts. JP is the principal investigator of the grant that funded the work presented in this manuscript; she was principally responsible for the research design and project management of the research grant that made this manuscript possible. She also participated in conducting interviews and editing drafts of this manuscript. All authors read and approved the final manuscript.

## Appendix: Interview guide and protocol notes

### Notes on interview protocol

Interviewers used the guide presented in Table 3  to conduct participant interviews, using a semi-structured format. Interviewers were not required to use the probes listed; these were provided as aids to facilitate the interviewer's task by illustrating the type of information for which the interviewers were to probe. Similarly, although the questions are listed in the suggested order, interviewers were free to change the order of the questions to better fit the flow of the interview.

Interviews were scheduled to be one hour in length, with one half-hour between interviews for interviewers to compile notes on the completed interview and conduct administrative tasks (e.g., labeling the interviews on the memory card, recording interviewee information in a participant record). In some cases, the interviews went somewhat over the one-hour mark, but never more than approximately 10 minutes. In a very few instances, the participants' comments were concise enough that the interview ended before the one-hour mark. However, most interviews lasted approximately one hour.

**Table 3:** Interview Guide

**CONCEPT TAPPED**	**PRIMARY QUESTION**	**POSSIBLE PROBES**
**Quality of Care in General**	1. How do you or your staff identify quality of care issues in need of improvement for your **OUTPATIENT **primary care clinics?	*Probe for explicit processes (e.g., strategic planning, balanced score cards, data that is monitored, etc.)*
		a. Who would be responsible for initiating and carrying out such efforts?
		b. Who would be responsible for monitoring such efforts?
**Mental Models of Clinical Practice Guidelines (CPG)**	2. What does the term "Clinical Practice Guidelines" mean to you?	a. What role do you see for clinical practice guideline use as a method for improving quality of care?
		b. Do you believe clinical practice guidelines are effective for improving quality of care? Please explain.
		If no, follow up with, "Despite your beliefs, what is your experience?
	3. How do guidelines help you improve the quality of care you provide your patients?	a. As a source of data feedback?
		b. How is data collected and utilized in your facility to improve the quality of patient care (e.g., administrative "scorekeeping" or as feedback for improving the quality of care)?
		c. Was EPRP data or other data on performance distributed?
		d. Did EPRP results affect individual performance evaluations?
		e. Does the facility collect clinical outcome data (mortality, readmission, functional status) related to the guideline?
**CPG Success Story**	4. Could you tell us the story of a time you and your team successfully implemented a clinical practice guideline (e.g., smoking cessation, depression screening, diabetes mellitus, hypertension, etc.)?	*Probe for the Who, What, When, Where, & How of the story*.
		a. What were the steps?
		b. Who was involved? To what extent are clinicians involved in determining how to implement guidelines?
		c. How was this guideline effort brought to the attention of clinicians and managers in your facility? (e.g., formal meetings, guideline champions, grand rounds, e-mail distributions, web sites, etc)?
		d. To what extent were committees (one steering committee for all guidelines or guideline specific committees) used to implement guidelines?
		e. What made it a success?
**CPG Training Development**	5. Please describe the training (i.e., professional development) that clinicians have received for implementing guidelines.	a. Would clinicians say they have been provided adequate support for professional development with respect to CPG implementation?
		b. Any training in the use of technology (e.g., CPRS, clinical reminders, etc.)?
		c. CME credit?
**Facilitators**	6. What are the most important factors that facilitate guideline implementation?	a. Technology (CPRS, clinical reminders)?
		b. Targeted educational or training programs, patient specific reminder systems, workshops, retreats?
		c. Incentives (e.g., monetary, extra time off from work, gift certificates, etc.)?
		d. Mentoring or coaching?
		e. Additional resources (e.g., equipment, staff, etc.)?
		f. Social Factors such as teamwork or networks?
		g. Representation from a diversity of service lines?
		h. Presence of a guideline champion?
		i. Supportive leadership (i.e., VISN and/or facility)?
		j. Pocket cards or "lite" versions of the guidelines?
**Barriers**	7. What are the most important factors that hinder guideline implementation?	a. Lack of resources or staff?
		b. Time (i.e., patient interactions are targeted for 20 minutes)?
		c. Lack of training?
		d. Not enough support?
		e. Financial?
**Innovations**	8. Were there any changes or redesigns in the clinical practices or equipment that supported the use of CPGs.	a. How were forms/procedures or reports changed to support adherence to guidelines?
		b. How were the responsibilities of nurses, aides, other personnel changed to support adherence?
		c. How were resources allocated/reallocated to support adherence?
**Structural, logistic, and organizational factors**	9. Please describe any other conditions that may influence CPG implementation?	a. Size of the facility?
		b. Academic affiliation?
		c. Competition with other QI initiatives?
		d. Location (e.g., remote vs. main facility)?
